# Detailed bone assessment of the sacroiliac joint in a prospective imaging study: comparison between computed tomography, zero echo time, and black bone magnetic resonance imaging

**DOI:** 10.1007/s00256-022-04097-3

**Published:** 2022-06-30

**Authors:** Lucas Wolharn, Roman Guggenberger, Kai Higashigaito, Thomas Sartoretti, Sebastian Winklhofer, Christine B. Chung, Tim Finkenstaedt

**Affiliations:** 1 Institute of Diagnostic and Interventional Radiology, University Hospital Zurich, University of Zurich, Zurich, Switzerland; 2 Faculty of Medicine, University of Zurich, Zurich, Switzerland; 3 Department of Neuroradiology, University Hospital Zurich, University of Zurich, Zurich, Switzerland; 4 Department of Radiology, University of California, San Diego, La Jolla, CA USA; 5 Department of Radiology, VA San Diego Healthcare System, San Diego, CA USA

**Keywords:** Spondylarthropathies, Sacroiliac joint, MRI, Zero echo time, Ultrashort echo time, UTE, ZTE, CT, Black bone, Diagnostic performance

## Abstract

**Objectives:**

To compare the value of zero echo time (ZTE) and gradient echo “black bone” (BB) MRI sequences for bone assessment of the sacroiliac joint (SI) using computed tomography (CT) as the reference standard.

**Materials and methods:**

Between May 2019 and January 2021, 79 patients prospectively underwent clinically indicated 3-T MRI including ZTE and BB imaging. Additionally, all patients underwent a CT scan covering the SI joints within 12 months of the MRI examination. Two blinded readers performed bone assessment by grading each side of each SI joint qualitatively in terms of seven features (osteophytes, subchondral sclerosis, erosions, ankylosis, joint irregularity, joint widening, and gas in the SI joint) using a 4-point Likert scale (0 = no changes–3 = marked changes). Scores were compared between all three imaging modalities.

**Results:**

Interreader agreement was largely good (*k* values: 0.5–0.83). Except for the feature “gas in SI joint” where ZTE exhibited significantly lower scores than CT (*p* < 0.001), ZTE and BB showed similar performance relative to CT for all other features (*p* > 0.52) with inter-modality agreement being substantial to almost perfect (Krippendorff’s alpha coefficients: 0.724–0.983). When combining the data from all features except for gas in the SI joint and when binarizing grading scores, combined sensitivity/specificity was 76.7%/98.6% for ZTE and 80.8%/99.1% for BB, respectively, compared to CT.

**Conclusions:**

The performance of ZTE and BB sequences was comparable to CT for bone assessment of the SI joint. These sequences may potentially serve as an alternative to CT yet without involving exposure to ionizing radiation.

## Introduction

Changes in the sacroiliac (SI) joints are frequently related to degenerative disease, secondary to rheumatologic (e.g., axial spondyloarthropathies) or other nonrheumatologic disorders such as infectious, drug-, trauma-, or pregnancy-related [1]. Imaging findings of the SI joints are important since clinical decision-making heavily relies on these findings. Notably, in the context of seronegative spondyloarthropathies, imaging findings are particularly important as these diseases are not diagnosed using specific biochemical markers. Furthermore, imaging findings are included in the Assessment of Spondyloarthritis (ASAS) axial Spondyloarthritis (axSpA) classification system [2]. Although MRI is key for the detection of early changes like bone marrow edema, osteitis, enthesitis, or capsulitis [3], CT and conventional radiography can better identify specific structural osseous changes of the SI joints occurring at a later stage of the disease, e.g., erosions, subcortical sclerosis, and ankylosis, and help to differentiate post-inflammatory from degenerative changes [4]. Early diagnosis of these osseous changes is of paramount importance to correctly diagnose patients, prevent therapeutic delays, and improve overall outcome.

**Fig. 1:**
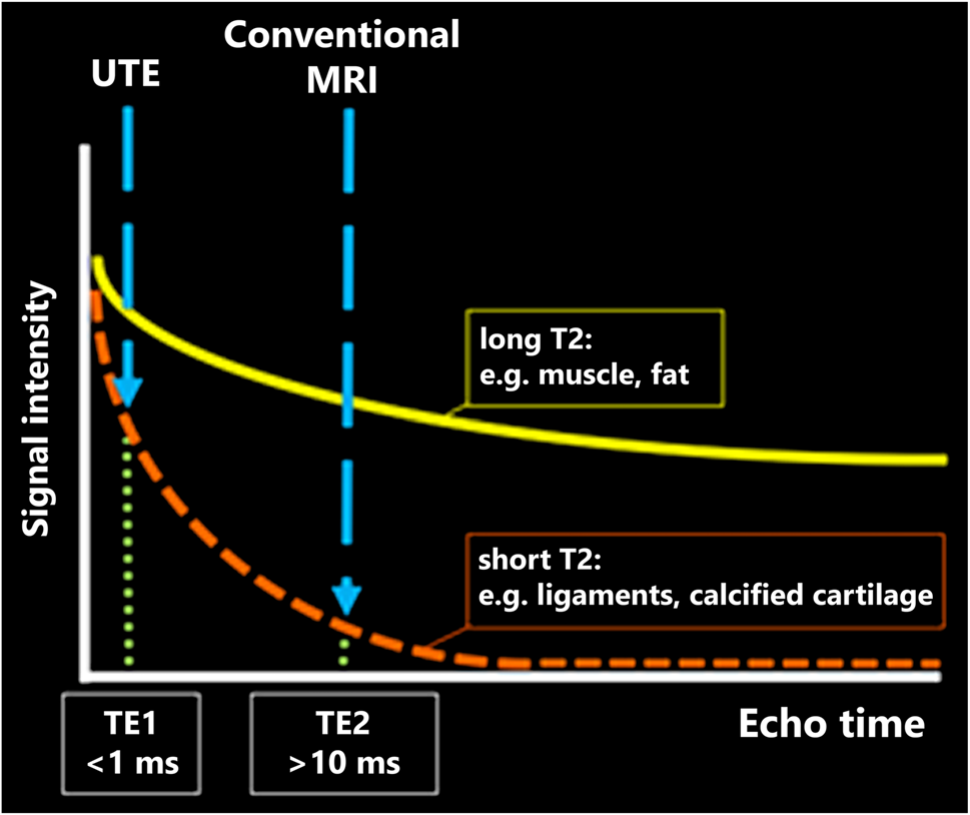
The decay of MR signal in tissues with different T2 properties is shown. In contrast to long T2 tissues like muscle and fat, in short T2 tissues like ligaments and calcified cartilage, the signal decays more rapidly. Using zero echo time and ultrashort echo time sequences featuring a very short TE (< 1 ms echo time), sufficient signal can be acquired. Abbreviations: TE = echo time; UTE = ultrashort echo time; MRI = magnetic resonance imaging

Recently, new MRI sequences have become available that may allow for accurate cortical and trabecular bone assessment. ZTE (zero echo time) [5–7] and UTE (ultrashort echo time) [8–11] MRI sequences belong to a group of novel 3D MRI pulse sequences that use an echo time (TE) of 1 ms or less enabling the acquisition of sufficient signal from rapidly decaying short-T2 tissue components which are abundant in calcified cartilage such as at the transition of articular cartilage to the subchondral bone, tendons, and menisci (Fig. 1) [12–14].

In particular, in these short echo time sequences, signal can be provided from short T2 tissues to provide a contrast mechanism that affords visualization of the cortical bone of the SI joint [5, 6, 12]. Importantly, ZTE and UTE feature comparable contrast-to-noise and signal-to-noise ratios (CNR/SNR) [15].

Another promising established MRI sequence that may potentially enable bone assessment is the black bone (BB) sequence. This 3D low flip angle gradient-echo MRI sequence features high contrast between bone and other tissues while providing a low contrast between those tissues [16–18].

As previously described, it would be desirable to detect both early (e.g., bone edema) and more chronic (subchondral sclerosis) changes in the SI joints by a single MRI scan as an “all-in-one” diagnostic step. A holistic MRI protocol would be more time and cost-efficient, optimizes the diagnostic workflows, and would potentially even allow for the replacement of CT imaging in certain circumstances reducing healthcare costs and ionizing radiation exposure and allowing for more efficient and timely clinical interpretation. Though low-dose CT is broadly implemented, the ALARA (as low as reasonably achievable) principles should also be considered with avoidance of any ionizing radiation if possible. Especially, since younger patients are predominately affected by rheumatological changes in the SI joint. Thus, the goal of this prospective study was to evaluate the diagnostic performance of ZTE and black bone MRI sequences for bone assessment of the SI joint and demonstrate the non-inferiority compared to CT as the reference standard.

There is no prior work, to our knowledge, that has explored the value of ZTE and BB MRI sequences intra-individually for the osseous assessment of the SI joints compared to CT as the reference standard.

## Materials and methods

### Study design

This prospective study was approved by the cantonal ethics committee of Zurich, and all participants provided written informed consent for study participation, collection of data, and use of prototype pulse sequences.

### Participants

Patients referred to our department for a clinically indicated MRI scan between May 2019 and January 2021 were enrolled in our study. All patients considered for enrollment had undergone a clinically indicated CT scan covering the SI joints within 12 months of their MRI examination (the average time gap between the CT and MRI scan was 5.2 ± 5.4 months). If patients agreed to participate, two additional study sequences (i.e., ZTE and BB) were added to the respective standard MRI examination.

Exclusion criteria were any form of incomplete datasets or major artifacts due to motion or foreign bodies.

### MR imaging

All MRI scans were performed on a 3-T scanner “GE Healthcare Discovery 750 W plus GEM” (GE Healthcare, Chicago, IL, USA) using an abdomen coil. All patients were positioned feet first and supine. Sequence characteristics for ZTE and BB are summarized in Table 1. Both sequences are developed by General Electrics and commercially available under the names of ZTE and LAVA FLEX. ZTE images were acquired in axial and BB images in a coronal plane in isotropic resolution and hence were used for multiplanar reconstructions with a slice thickness and increment of 2 mm, each, in all three planes. The typical through-plane coverage was 250 slices for ZTE and 400 slices for BB, ranging from the 12th thoracic vertebra to the lesser trochanter. Acquisition was accelerated 2 × using ARC (Autocalibrating Reconstruction for Cartesian imaging). No semicoronal reformations were performed for ZTE and BB sequences, because there were no adequate CT datasets for the custom semicoronal reformations available for the majority of CT scans. There was no readout for the SI joints on standard MRI sequences to show the additional value of ZTE and BB since the clinical MRI protocol was not standardized.

**Table 1:** MR sequence characteristics for ZTE (zero echo time) and BB (black bone)

Sequence	TR (ms)	TE (ms)	Acquisition matrix	Reconstruction matrix	Slice thickness (mm)	FOV (cm)	BW (kHz)	In-plane acceleration factor	FA (°)	Voxel dimensions (xyz, mm)	Scan time (min)
ZTE	5.1	≅ 0	212 × 212 × 250	256 × 256 × 250	1.5	32	± 62.5	**/**	2	1.5 × 1.5 × 1.5	4:06
BB	5.1	1.2	340 × 340 × 200	512 × 512 × 400	1	39	± 125	ARC (2 ×)	5	1.1 × 1.1 × 1	3:46

*ZTE*, zero echo time; *BB*, black bone; *TR*, time to repetition; *TE*, echo time; *FOV*, field of view; *BW*, bandwidth; *ARC*, Autocalibrating Reconstruction for Cartesian imaging; *FA*, flip angle.

### CT imaging

Patients were included if the sacroiliac joints were covered in the CT examination. The majority of scans consisted of an abdominal or pelvic CT scan performed on a second-generation energy-integrating detector dual-source CT scanner (Siemens Somatom Definition Flash, Siemens Healthineers, Erlangen, Germany) using the following characteristics: tube voltage of 90, 100, 110, and 120 kVp; tube current: 100–150 mAs (with active tube modulation); field-of-view (FOV) of 500 mm with a matrix of 512 × 512; and a bone kernel (mostly Br59) for axial image reconstruction. Image data was then reformatted in all three planes with a slice thickness and increment of 2 mm, each.

### Assessment of osseous sacroiliac joint changes

**Fig. 2:**
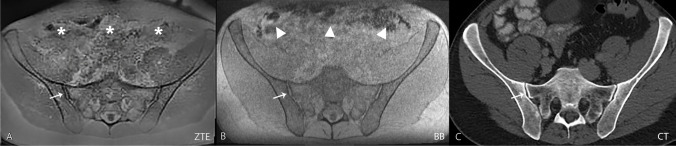
In this 42-year-old male patient referred for a kidney pathology, the SI joints were rated normal. Transverse images of ZTE (image **A**), BB (image **B**), and CT (image **C**) of the pelvis are shown without any pathologic findings in the SI joint. The cortical contour of the SI joint (thin arrows) is well-delineated in all three images. The ZTE image features a “flat soft tissue contrast” and thus emphasizes the bony contours of the pelvis. Gas in the bowel is more apparent in the black bone gradient echo (triangles) sequence compared to the ZTE sequence (asterisks). Abbreviations: SI = sacroiliac joint; ZTE = zero echo time; BB = black bone; CT = computed tomography

The assessment was performed independently by two board-certified radiologists with 9 and 10 years of experience in musculoskeletal imaging. The readout of the two datasets (ZTE and BB; Fig. 2) was conducted in two different sessions, separated by 2 weeks and in a random order to avoid recall bias. Both readers were blinded to patient identification and clinical data as well as to the results of the other datasets.

The changes in the SI joints were assessed using a 4-point Likert scale (0–3, 0 = no changes, 1 = subtle changes, 2 = moderate changes, 3 = marked changes) for each of the seven different features (osteophytes, subchondral sclerosis, erosions, ankylosis, joint irregularity, joint widening, and gas in the SI joints).

The CT scan served as the reference standard for the bone assessment of the SI joints. To obtain a reference standard, a consensus readout on the corresponding CT datasets was performed 2 weeks after the last MRI readout session by the two readers. Both readers were blinded to their previous ratings for the MRI ZTE and BB datasets during the consensus readout.

Each SI joint of each patient was evaluated individually, resulting in a total sample size of 178.

### Statistics

The Cohen *k* test was performed to evaluate the inter-reader agreement regarding the qualitative evaluation of the SI joints: *k* values of less than 0.20 were indicative of poor agreement; 0.21–0.40, fair agreement; 0.41–0.60, moderate agreement; 0.61–0.80, good agreement; and 0.81–1.00, excellent agreement, according to the method of Landis and Koch [19].

Data distribution was checked qualitatively (histograms, boxplots, quantile–quantile plots) and quantitatively (Shapiro-Wilks tests). Differences between the imaging modalities in terms of scores were assessed by Friedman tests followed by post-hoc signed-rank tests. Additionally, Krippendorff’s alpha coefficients were computed to assess the agreement of scores between ZTE and CT as well as between BB and CT (alpha coefficients were interpreted with the same scale as described above). Eventually, scores were binarized so that scores of 0 and 1 indicated the absence of a pathologic finding, and scores of 2 and 3 indicated the presence of a pathologic finding. Subsequently, using CT as the reference standard, diagnostic accuracy parameters were computed for ZTE and BB sequences. *p*-values were adjusted for multiple comparisons using the Benjamini–Hochberg procedure. A two-sided *p*-value of less than 0.05 was considered significant. All analyses were performed in the R programming language for statistical computing (version 4.0.2) (R Core Team, 2020). All statistics were calculated by the two co-authors S.W. and T.S.

## Results

Ninety-seven patients were initially included in the study and examined by 3-Tesla MRI. Eight patients were excluded due to incomplete ZTE/BB or CT datasets or poor image quality due to motion artifacts. Eventually, 89 patients (55 men and 34 women with a mean age of 55.7, standard deviation of 15 years, and median of 57 years) were enrolled in the final analysis. The average body mass index (BMI) was 25.8 with a standard deviation of 3.1 kg/m^2^. The mean time gap between the CT and MRI scan was 5.2 months (with a standard deviation of 5.4 months, a median of 4 months, a minimum of 0, and a maximum of 12 months).

**Fig. 3:**
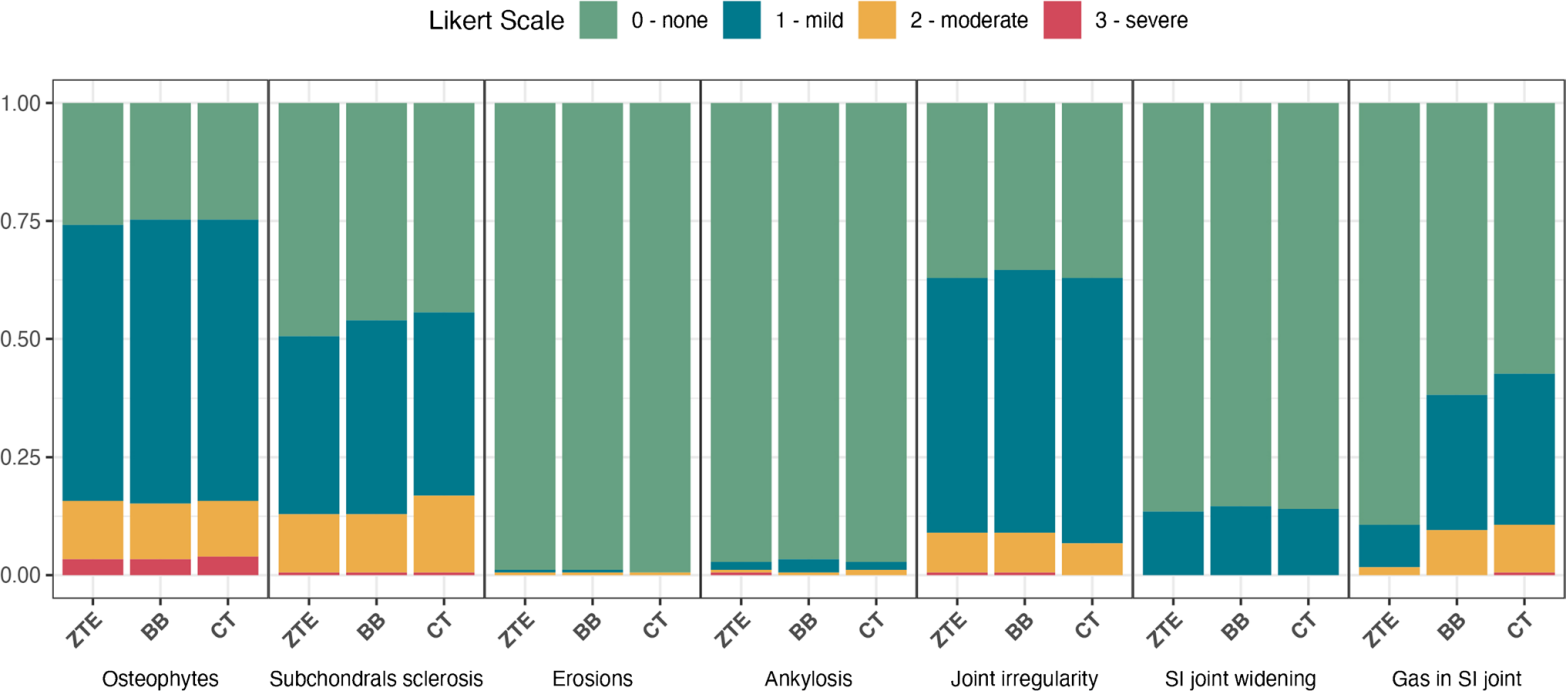
Graphic overview of scores derived from image assessment. The data is visualized using bar plots. Note that the scores for each imaging modality across all assessment categories were comparable except for the category “gas in SI joint.” In this category, ZTE had lower scores than BB and CT. Abbreviations: ZTE = zero echo time; BB = black bone; CT = computed tomography; SI = sacroiliac joint

**Fig. 4:**
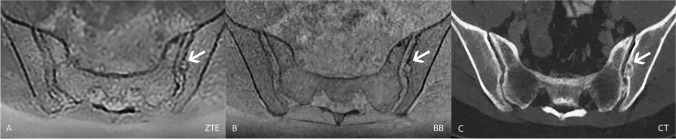
In this 48-year-old female patient referred for a workup of a bowel pathology, erosions were detected on all three transverse images. The erosion (arrow) appeared to be chronic since there was a sclerotic rim visible on the ZTE (image **A**) and BB (image **B**) sequence as well as on the CT scan (image **C**). Abbreviations: ZTE = zero echo time; BB = black bone; CT = computed tomography; SI = sacroiliac joint

**Fig. 5:**
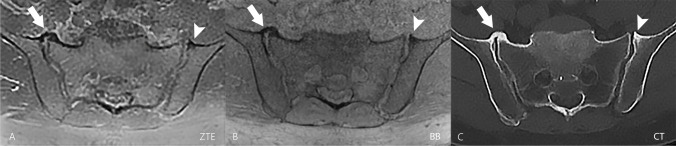
This 72-year-old female patient was referred for tumor staging. The transverse ZTE image (**A**) showed right-sided bridging osteophytes of the anterosuperior sacroiliac joint (arrowhead) and bone spurs on the left side (arrow) that were also perfectly visible on the BB sequence (**B**) and the CT scan (**C**). In this patient, the signal-to-noise ratio was lower in the ZTE and BB sequence since the patient had a high body mass index of 29.4. In comparison, for the CT scan, the required optimal dose to ensure sufficient image quality was tailored to the individual patient by the modern CT scanner. However, the image quality of the ZTE and BB sequence is still sufficient to characterize the SI joint bone findings. Abbreviations: ZTE = zero echo time; BB = black bone; CT = computed tomography; SI = sacroiliac joint

**Fig. 6:**
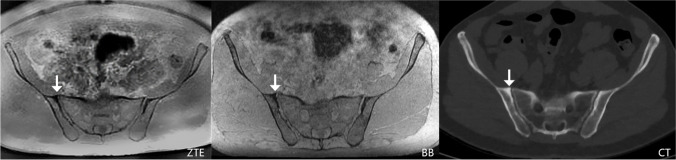
This 62-year-old female patient was referred for liver pathology workup and demonstrated subchondral sclerosis on the right ilium, adjacent to the SI joint (arrow). Note the high contrast between sclerotic and trabecular bone on ZTE and CT as well as reduced “blooming” artifacts of bowel gas on ZTE compared to BB. Abbreviations: ZTE = zero echo time; BB = black bone; CT = computed tomography; SI = sacroiliac joint

A detailed overview of the data is provided in Fig. 3 and Table 2. Inter-reader agreement was moderate to excellent (*k* values: 0.5–0.83). Except for the feature gas in SI joints, there were no significant differences between the three imaging modalities for the remaining 6 features (*p* > 0.52 for all 6 features, Figs. 4, 5, and 6). For the feature gas in SI joints, ZTE exhibited significantly lower scores than both BB and CT (both *p* < 0.001), while there were no significant differences between BB and CT (*p* = 0.1).

**Table 2:** Overview of the Likert scores derived from the consensus readout of the three datasets (ZTE, BB, and CT). Please note the low prevalence of erosions and ankylosis in our patient collective

ZTE (*n* = 178)							
Likert scale	Osteophytes	Sclerosis	Erosions	Ankylosis	Irregularity	Widening	Air
0	46	88	176	173	66	154	159
1	104	67	1	3	96	24	16
2	22	22	1	1	15	0	3
3	5	1	0	1	1	0	0
BB** (*n* = 178)**							
Likert scale	Osteophytes	Sclerosis	Erosions	Ankylosis	Irregularity	Widening	Air
0	44	82	176	172	63	152	110
1	107	73	1	5	99	26	51
2	21	22	1	1	15	0	17
3	5	1	0	0	1	0	0
CT** (*n* = 178)**							
Likert scale	Osteophytes	Sclerosis	Erosions	Ankylosis	Irregularity	Widening	Air
0	44	79	177	173	66	153	102
1	106	69	0	3	100	25	57
2	21	29	1	2	12	0	18
3	6	1	0	0	0	0	1

ZTE, zero echo time; BB, black bone; CT, computed tomography.

Furthermore, except for the feature gas in SI joints on ZTE (alpha = 0.179), Krippendorff’s alpha coefficients indicated a substantial or almost perfect agreement for all other features of both MRI sequences (ZTE and BB) compared to CT. Lastly, when combining the diagnostic performance of all parameters, ZTE achieved a sensitivity and specificity of 64.1% and 98.8%, respectively, while BB achieved a sensitivity of 78.3% and a specificity of 98.9% compared to CT. If the single feature “gas in SI joints” was excluded from the calculation of the diagnostic performance, there was an improvement particularly for ZTE with 76.7%/98.6% sensitivity/specificity, while BB achieved 80.8%/99.1% sensitivity/specificity compared to CT. Sensitivity and specificity are listed in Table 3.

**Table 3:** Data overview of the various measures of diagnostic accuracy, as well as inter-modality agreement of scores with their respective confidence intervals

	ZTE			BB		
	Sensitivity	Specificity	Krippendorff’s alpha	Sensitivity	Specificity	Krippendorff’s alpha
Osteophytes	0.893; [0.778, 1]	0.98; [0.958, 1]	0.923	0.929; [0.833, 1]	0.993; [0.98, 1]	0.983
Subchondral sclerosis	0.666; [0.498, 0.835]	0.98; [0.957, 1]	0.834	0.7; [0.536, 0.864]	0.986; [0.968, 1]	0.899
Erosions	1; [1]	1; [1]	0.669	1; [1]	1; [1]	0.669
Ankylosis	0.5; [1, 1]	0.994; [0.006, 0.983]	0.792	0.5; [1, 1]	1; [1]	0.724
Joint irregularity	0.75; [0.505, 0.995]	0.958; [0.927, 0.988]	0.847	0.833; [0.622, 1]	0.964; [0.935, 0.992]	0.881
Joint widening	1; [1]	1; [1]	0.882	1; [1]	1; [1]	0.977
Gas in the SI joint	0.158; [0, 0.322]	1; [1]	0.179	0.684; [0.475, 0.893]	0.975; [0.95, 0.999]	0.67
All features combined	0.641; [0.543, 0.739]	0.988; [0.982, 0.994]	0.827	0.783; [0.698, 0.867]	0.989; [0.983, 0.995]	0.91
All features except for gas in the SI joint	0.767; [0.67, 0.864]	0.986; [0.979, 0.993]	0.92	0.808; [0.718, 0.899]	0.991; [0.985, 0.997]	0.955

*ZTE*, zero echo time; *BB*, black bone; *SI*, sacroiliac joint.

## Discussion

Our study showed that a ZTE and BB sequence provides similar diagnostic performance to CT with a high inter-reader and inter-modality agreement for six of the seven investigated features (except for the feature “gas in the SI joints”). However, the low prevalence of two of the investigated features in our study group (erosions, 1/178 of SI joints and ankylosis, 2/178 of SI joints) limited the meaning of the calculated sensitivity and specificity values for these two features.

Adding a ZTE or BB sequence to the regular SI joints MRI protocol would allow for the simultaneous detection of both osseous and non-osseous (e.g., cartilage abnormalities and enthesitis) changes in the SI joints with only one single MRI scan. The results show the value of ZTE and BB MRI sequences in displaying precise osseous morphology and offering detail beyond what traditional MR sequences can provide. Streamlining the diagnostic process can improve time to treatment, especially critical in inflammatory etiologies.

Notably, scan time is prolonged only by approximately 4 min by adding a ZTE or BB sequence to the MRI protocol and due to the 3D nature of the datasets, customized multiplanar reformations can be performed.

Only in terms of the detection of gas in the SI joints, ZTE and to a lesser extent BB showed significantly lower sensitivity than CT. In general, the presence of gas in the SI joints is, analogous to other joints, most frequently considered to be a nonspecific finding or a sign of degeneration or recent trauma. This imaging finding corresponds to a vacuum phenomenon primarily caused by the intraarticular accumulation of nitrogen [20, 21]. Gas bubbles lead to susceptibility artifacts on MR images, to which ZTE sequences are less prone owing to a very low echo time and flip angle [22, 23]. On the other hand, due to the nature of gradient-echo MR sequences, gas accumulation becomes more apparent in the BB sequence [24].

Some of the different SI joint pathologies arise from etiologies (e.g., traumatic and rheumatic) which are present in younger patients where ionizing radiation is a particular concern. Especially, since radiosensitive organs, like the ovaries and testes, are located in close proximity to the SI joints [25], the substitution of a CT scan by ionizing radiation-free ZTE or BB MRI sequences would remedy this concern.

The combination of an image acquisition that is not associated with the exposure to ionizing radiation, precise bone depiction, and detection of early sacroiliac joint changes like bone marrow edema makes SI joint MRI augmented by a ZTE or BB sequence a plausible “all in one” diagnostic procedure and may render CT imaging for some assessments obsolete. This is in line with other MRI studies about ZTE in other body regions, e.g., the shoulder and the spine, where a successful evaluation of cortical and trabecular bone without CT has been demonstrated [7, 8, 26, 27].

Our study has limitations. First, the period between CT and MRI ranged from 0 to 12 months. However, the average time gap between the CT and MRI scan was only 5.2 ± 5.4 months. Within this short period, rapid changes to the SI joints in our random patient cohort were unlikely. Second, no analysis of the ZTE and BB sequences in the semicoronal planes was performed even though these are efficient planes for the detection of osseous SI joint changes, since there were no adequate CT datasets for the custom reformations available for the majority of scans. Third, to compute sensitivity and specificity, we elected to dichotomize the assessment scores and hence erased possible small differences in the grading of the two readers (between Likert scales of 0 and 1 or 2 and 3). Lastly, the prevalence of pathologic changes in the SI joints in our patient cohort was rather low and less distinct, because the included patients were randomly selected mirroring the general population as described in the “Materials and methods” section. Additional prospectively acquired studies dedicated to the SI joints, especially in patients with SI joint pathology (e.g., erosions), would be helpful to reconfirm the results of our study.

## Conclusion

Overall performance of the detailed bone assessment of the SI joints using ZTE and BB sequences was comparable to CT and hence may serve as an alternative to CT for the assessment of cortical and trabecular bone alterations.

## Abbreviations

BB: Black bone
CT: Computed tomography
MRI: Magnetic resonance imaging
TE: Echo time
TR: Repetition time
ZTE: Zero echo time
UTE: Ultrashort echo time
BMI: Body mass index
